# Observation of dark edge states in parity-time-symmetric quantum dynamics

**DOI:** 10.1093/nsr/nwad005

**Published:** 2023-01-10

**Authors:** Peng Xue, Xingze Qiu, Kunkun Wang, Barry C Sanders, Wei Yi

**Affiliations:** Beijing Computational Science Research Center, Beijing 100084, China; Key Laboratory of Quantum Information, University of Science and Technology of China, Chinese Academy of Sciences, Hefei 230026, China; Synergetic Innovation Center in Quantum Information and Quantum Physics, University of Science and Technology of China, Chinese Academy of Sciences, Hefei 230026, China; School of Physics and Optoelectronic Engineering, Anhui University, Hefei 230601, China; Shanghai Branch, National Laboratory for Physical Sciences at Microscale, University of Science and Technology of China, Shanghai 201315, China; Institute for Quantum Science and Technology, University of Calgary, Alberta T2N 1N4, Canada; Program in Quantum Information Science, Canadian Institute for Advanced Research,Toronto, M5G 1Z8, Canada; Key Laboratory of Quantum Information, University of Science and Technology of China, Chinese Academy of Sciences, Hefei 230026, China; Synergetic Innovation Center in Quantum Information and Quantum Physics, University of Science and Technology of China, Chinese Academy of Sciences, Hefei 230026, China

**Keywords:** parity-time symmetry, photonic quantum walks, dark edge states, topological invariants

## Abstract

Topological edge states arise in non-Hermitian parity-time ($\mathcal {PT}$)-symmetric systems, and manifest themselves as bright or dark edge states, depending on the imaginary components of their eigenenergies. As the spatial probabilities of dark edge states are suppressed during the non-unitary dynamics, it is a challenge to observe them experimentally. Here we report the experimental detection of dark edge states in photonic quantum walks with spontaneously broken $\mathcal {PT}$ symmetry, thus providing a complete description of the topological phenomena therein. We experimentally confirm that the global Berry phase in $\mathcal {PT}$-symmetric quantum-walk dynamics unambiguously defines topological invariants of the system in both the $\mathcal {PT}$-symmetry-unbroken and -broken regimes. Our results establish a unified framework for characterizing topology in $\mathcal {PT}$-symmetric quantum-walk dynamics, and provide a useful method to observe topological phenomena in $\mathcal {PT}$-symmetric non-Hermitian systems in general.

## Teaser

Dark edge states are detected experimentally for the first time in photonic quantum walks with spontaneously broken parity-time symmetry.

## INTRODUCTION

Topological phases exhibit remarkable properties and challenge our understanding of phases and phase transitions [1–7]. Instead of local order parameters, such phases are characterized by non-local topological invariants, which dictate the existence and number of topological edge states at an interface through the bulk-boundary correspondence [8–14]. Photonic quantum walks (QWs) [15–22] offer a versatile platform on which topological phenomena can be simulated and studied in quantum dynamics [23–29]. Because of the ease of introducing loss, photonic QWs allow the exploration of topological phenomena in the context of non-unitary dynamics [30–35]. Recent experimental observations of topological edge states in parity-time ($\mathcal {PT}$)-symmetric systems have stimulated effort in clarifying the relation between topology and $\mathcal {PT}$ symmetry [35–40]. Previous experiments have probed topological invariants and edge states in the $\mathcal {PT}$-symmetry-unbroken regime [35–43], where eigenenergies of the $\mathcal {PT}$-symmetric non-Hermitian Hamiltonian are entirely real [44–59]. In the $\mathcal {PT}$-symmetry-broken regime where eigenenergies become complex, the definition of topological invariants and the detection of topological edge states can be elusive [60–62]. In particular, in the $\mathcal {PT}$-symmetry-broken regime, topological edge states are $\mathcal {PT}$-symmetry broken, and can be classified as bright and dark edge states [36,41], depending on their eigenenergies. Whereas experimental signals of bright edge states can be probed from enhanced local probabilities at the boundary, local signals of dark edge states are easily overwhelmed by those of the bulk states, and are therefore experimentally elusive.

In this work we experimentally detect both types of edge states (dark edge states in particular), and experimentally confirm that the global Berry phase in non-unitary QW dynamics gives rise to well-defined topological invariants in both the $\mathcal {PT}$-symmetry-unbroken and -broken regimes, and is responsible for the emergence of both types of topological edge states. For the detection of dark edge states, we resort to the discrete-time evolution of the integrated probability distribution, rather than local signals.

## RESULTS

### $\mathcal {PT}$ -symmetric QWs

We consider discrete-time QWs, where an operator $\tilde{U}^{\prime }$ repeatedly acts upon the walker state, leading to periodically driven Floquet dynamics. The Floquet operator $\tilde{U}^{\prime }$ is hence central to the description of QWs. For the $\mathcal {PT}$-symmetric non-unitary QWs studied here, we focus on the operator


(1) \begin{eqnarray*} \tilde{U}^{\prime }&=&R\bigg (\frac{\theta _1}{2}\bigg )SR \bigg (\frac{\theta _2}{2}\bigg )\tilde{M}R\bigg (\frac{\theta _2}{2}\bigg )\\ &&SR \bigg (\frac{\theta _1}{2}\bigg ). \end{eqnarray*}

Here the QW is on a one-dimensional integer lattice $\mathbb {L}$ on a circle, with site index −*N* ≤ *x* ≤ *N* and *N* being the largest positive site index. The conditional-shift operator $S=\sum _x(\mathinner {|{x-1}\rangle }\mathinner {\langle {x}|}\otimes \mathinner {|{0}\rangle }\mathinner {\langle {0}|}+\mathinner {|{x+1}\rangle }\mathinner {\langle {x}|}\otimes \mathinner {|{1}\rangle }\mathinner {\langle {1}|})$ moves the walker in the two orthogonal coin states |0〉 and |1〉 to the left and right by one lattice site, respectively (see the online supplementary material). The position-dependent coin operator $R(\theta ) = {\mathbb{1}}_{\rm w}\,\,{\otimes}\,\, e^{-i\theta \sigma _y}$ rotates the coin state by position-dependent θ about the *y* axis, where $\mathbb {1}_{\rm w}=\sum _{\mathbb {L}}\mathinner {|{x}\rangle }\mathinner {\langle {x}|}$. Non-unitarity is enforced by the gain-loss operator


(2) \begin{eqnarray*} \tilde{M} &=& \gamma \mathbb {1}_{\rm w}{\otimes} (\mathinner {|{+}\rangle }\mathinner {\langle {+}|}+ \sqrt{1-p}\mathinner {|{-}\rangle }\mathinner {\langle {-}|}),\\ &&\qquad 0<p\leqslant 1, \end{eqnarray*}

where $\mathinner {|{\pm }\rangle }=(\mathinner {|{0}\rangle }\pm \mathinner {|{1}\rangle })/\sqrt{2}$ and the gain-loss parameter γ = (1 − *p*)^−1/4^ with loss parameter *p* ∈ [0, 1]. Under $\tilde{M}$, states in | ± 〉 are amplified or suppressed by γ^±1^ in each step. Note that the decomposition of $\tilde{U}^{\prime }$ into the various operators is natural in the context of photonic QWs, as they correspond to gate operations on photons, as we discuss below. In particular, the loss parameter *p* and the position-dependent angles θ_1_ and θ_2_ are tunable through different optical components.

The non-unitary operator $\tilde{U}^{\prime }$ is $\mathcal {PT}$-symmetric as long as the coin parameters satisfy θ_1, 2_(*x*) = θ_1, 2_(*N* − *x*) under the periodic boundary condition. The symmetry operator is $\mathcal {PT}=\sum _{x\in \mathbb {L}}|x\rangle \langle N-x|\otimes \sigma _z\mathcal {K}$ with $\mathcal {PT}\tilde{U}^{\prime }(\mathcal {PT})^{-1}=\tilde{U}^{\prime -1}$, where $\mathcal {K}$ is complex conjugation. We note that $\tilde{U}^{\prime }$ is different from the previous $\mathcal {PT}$-symmetric case [35] for a much simpler gain-loss mechanism. Furthermore, $\tilde{U}^{\prime }$ is also different from the Floquet operator in [31], which has no explicit $\mathcal {PT}$ symmetry.

The non-unitary QW dynamics can be equivalently regarded as generated by the effective non-Hermitian Hamiltonian *H*_eff_ (see the Methods section for its explicit form), where $\tilde{U}^{\prime }=\exp (-i H_{\rm eff})$. The quasienergy ε of *H*_eff_ is defined through


(3) \begin{eqnarray*} \tilde{U}^{\prime }|\psi _{\lambda }\rangle =\lambda |\psi _{\lambda }\rangle , \qquad \lambda = e^{-i\epsilon }. \end{eqnarray*}

In the $\mathcal {PT}$-symmetry-unbroken regime, all {|ψ_λ_〉} are eigenstates of the $\mathcal {PT}$-symmetry operator, with {ε} being real and |λ| = 1. Otherwise, when the system has spontaneously broken $\mathcal {PT}$ symmetry, some ε become complex as |λ| ≠ 1. With varying coin parameters, the system can change from the $\mathcal {PT}$-symmetry-unbroken regime to the broken regime, where the transition is signaled by the first emergence of ε = 0 or ε = π in the quasienergy spectrum. We can further divide the $\mathcal {PT}$-symmetry-broken regime into partially broken and completely broken regimes, with {ε} being purely imaginary in the latter (see the online supplementary material).

**Figure 1.**
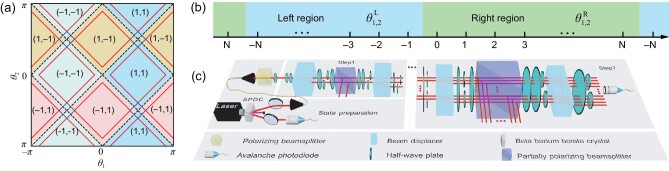
Phase diagram and experimental setup. (a) Phase diagram for $\mathcal {PT}$-symmetric non-unitary QWs governed by $\tilde{U}^{\prime }$, with coin parameters (θ_1_, θ_2_) and corresponding topological numbers (ν_0_, ν_π_). Dashed black lines represent topological phase boundaries. Solid red lines represent boundaries between $\mathcal {PT}$-symmetry-unbroken and -broken regimes, with $\mathcal {PT}$-symmetry-broken regimes lying in between the red lines near topological phase boundaries. Solid blue squares represent regimes with completely broken $\mathcal {PT}$ symmetry, where the eigenspectra are purely imaginary. (b) Left (*x* < 0) and right (*x* ≥ 0) regions for the $\mathcal {PT}$-symmetric QW. (c) Experimental setup for $\mathcal {PT}$-symmetric QWs with alternating losses. The photon pair is created via spontaneous parametric down-conversion (SPDC). One photon serves as a trigger. The other photon is projected into the polarization state $\mathinner {|{\pm }\rangle }$ (or $(\mathinner {|{+}\rangle }+i\mathinner {|{-}\rangle })/\sqrt{2}$) and then proceeds through the quantum-walk interferometric network. Finally, the photon is detected by an avalanche photodiode (APD), in coincidence with the trigger one. Photon counts give measured probabilities after correcting for relative efficiencies of the different APDs.

The non-unitary operator $\tilde{U}^{\prime }$ gives rise to Floquet topological phases (FTPs) in the dynamics, which are ensured by pseudo-anti-unitarity [63], with $\eta \tilde{U}^{\prime \dagger }\eta =\tilde{U}^{\prime }$ and $\eta =\sum _{x\in \mathbb {L}}|x\rangle \langle x|\otimes \sigma _x$. Here the σ_*j*_ (*j* = *x, y, z*) are Pauli matrices. We define topological invariants for these FTPs through the global Berry phase [60–62], which characterizes topological properties for both $\mathcal {PT}$-symmetry-unbroken and -broken regimes. As illustrated in Fig. 1(a), different topological phases are labeled by distinct topological numbers (ν_0_, ν_π_), whereas $\mathcal {PT}$ symmetry is spontaneously broken in the vicinity of topological phase boundaries.

As we detail in the online supplementary material, topological invariants defined through the global Berry phase are equivalent to winding numbers [36,37,41,42] or generalized Zak phases [35,43] in the $\mathcal {PT}$-symmetry-unbroken regime. Conversely, in the $\mathcal {PT}$-symmetry-broken regime, whereas generalized winding numbers and Zak phases become ill defined due to the emergence of ε = 0 or ε = π in the spectrum, the global Berry phases remain well defined and yield topological numbers that dictate the number of topological edge states.

To investigate topological edge states, we consider an inhomogeneous configuration, where interfaces exist near *x* = 0 and *x* = ±*N* as coin parameters on the left $(\theta ^{\text{L}}_1,\theta ^{\text{L}}_2)$ that are different from those on the right $(\theta ^{\text{R}}_1,\theta ^{\text{R}}_2)$. Depending on the coin parameters, topological edge states can emerge at interfaces near *x* = 0 and *x* = ±*N*, with the number of edge states having Re(ε) = 0 [Re(ε) = π] equal to the difference in the topological number ν_0_ (ν_π_) on either side of the boundary.

We analytically solve wave functions for topological edge states localized near *x* = 0. Interestingly, spatial wave functions of topological edge states are determined by coin parameters and are independent of γ. However, these edge states break $\mathcal {PT}$ symmetry, such that their quasienergies ε are complex with λ = ±γ or λ = ±1/γ. We identify those with λ = ±γ as bright edge states, with quasienergies ε = *i*ln γ and ε = π + *i *ln γ, respectively. As norms of the bright edge states scale as γ^2*t*^ in the *t*th step of the time evolution, local probabilities near the boundary would be amplified so long as the initial state has a finite overlap with the bright edge state. Conversely, edge states with λ = ±1/γ are identified as dark edge states, with quasienergies ε = −*i *ln γ and ε = π − *i *ln γ, respectively. Whereas dark edge states should give rise to a decay of local probabilities, such a decay is difficult to probe directly, as it is overwhelmed during the time evolution by the probability distribution of bulk states.

We note that, due to $\mathcal {PT}$ symmetry of $\tilde{U}^{\prime }$, bright and dark edge states necessarily emerge in pairs, albeit at different interfaces: a bright edge state near *x* = 0 is accompanied by a dark one near *x* = ±*N*, and a bright edge state near *x* = 0 becomes its dark counterpart after exchanging coin parameters of the left and right regions.

### Experimental detection of bright and dark edge states

As illustrated in Fig. 1, we use a photonic setup to implement a passive $\mathcal {PT}$-symmetric QW of single photons. The coin states |0〉 and |1〉 are respectively encoded in the horizontal $\mathinner {|{H}\rangle }$ and vertical $\mathinner {|{V}\rangle }$ polarizations of photons, whose spatial modes represent the lattice degrees of freedom. In our experiment, the initial coin state is prepared in either $\mathinner {|{\pm }\rangle }$ or $(\mathinner {|{+}\rangle }+i\mathinner {|{-}\rangle })/\sqrt{2}$, while the walker always starts from *x* = 0. We implement the coin-rotation operator *R*(θ), the shift operator *S* and the loss operator *M* using appropriate combinations of half-wave plates (HWPs), beam displacers and partially polarizing beamsplitters (PPBSs). The loss parameter *p* is fixed at 9/25 or 2/3, which is achieved using a PPBS with a certain polarization-dependent transmissivity. The experimentally realized time-evolution operator *U*′ differs from $\tilde{U}^{\prime }$ by a factor γ, since $\tilde{M}=\gamma M$. We therefore multiply the measured raw probability distribution *P*_R_(*x, t*) by a time-dependent scaling factor γ^2*t*^, so that the resulting corrected probability distribution *P*_C_(*x, t*) corresponds to $\mathcal {PT}$-symmetric QWs governed by $\tilde{U}^{\prime }$. For future reference, we define the normalized probability at the *t-*th step *P*_N_(*x, t*) = *P*_R_(*x, t*)/∑_*x*_*P*_R_(*x, t*).

We confirm the validity of topological invariants defined through the global Berry phase by detecting the bright topological edge states. As bright edge states feature amplified local probabilities, they are easily detected by monitoring the corrected probability near the boundary.

**Figure 2.**
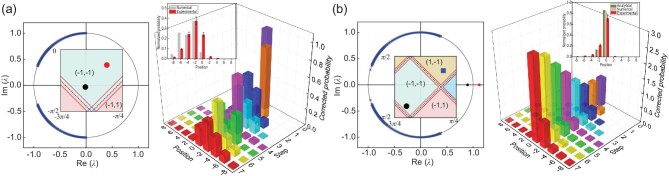
Experimental results. (a and b) Experimental observation of topological edge states in the $\mathcal {PT}$-symmetry-unbroken regime. We consider QWs with fixed *p* = 9/25 and $(\theta ^{\rm R}_1,\theta ^{\rm R}_2)=(-9\pi /16,-5\pi /16)$ in the right region, but with different coin parameters in the left region: $(\theta ^{\text{L}}_1,\theta _2^{\text{L}})=(-3\pi /8,-\pi /8)$ (a) and (π/16, 5π/16) (b). Initial state of the walker-coin system is $\mathinner {|{0}\rangle }{\otimes} \mathinner {|{+}\rangle }$. Left column: eigenvalues λ in the complex plane, where red and black dots represent eigenvalues of bright and dark edge states, respectively. Blue crosses represent eigenvalues of bulk states. Right column: measured corrected probability distributions up to seven steps. Inset: comparison between the measured (red) and numerically calculated (gray) normalized probability distribution at the seventh step, as well as that calculated from the analytical edge-state wave functions (cyan). Experimental errors are due to photon-counting statistics and represent the corresponding standard deviations.

We first examine the case where all coin parameters are chosen in the $\mathcal {PT}$-symmetry-unbroken regime. Without loss of generality, we fix the coin parameters in the right region with topological numbers (ν_0_, ν_π_) = ( − 1, −1), and vary coin parameters of the left region. When topological numbers are the same on either side of the boundary [Fig. 2(a)], the measured corrected probability distribution *P*_C_(*x, t*) up to seven steps shows no appreciable enhancement near the boundary *x* = 0, suggesting the absence of edge states. In contrast, when topological numbers of the left region are changed to (ν_0_, ν_π_) = (1, −1) [Fig. 2(b)], topological edge states should emerge at Re(ε) = 0. This is confirmed by the enhancement of the measured corrected probability *P*_C_(*x* = 0) near the boundary. Compared to the unitary QW, the corrected probability *P*_C_(*x* = 0) increases with the number of steps, which is a signature of bright edge states. The measured normalized spatial probability distribution *P*_N_(*x, t* = 7) agrees well with that calculated from analytical edge-state wave functions.

**Figure 3.**
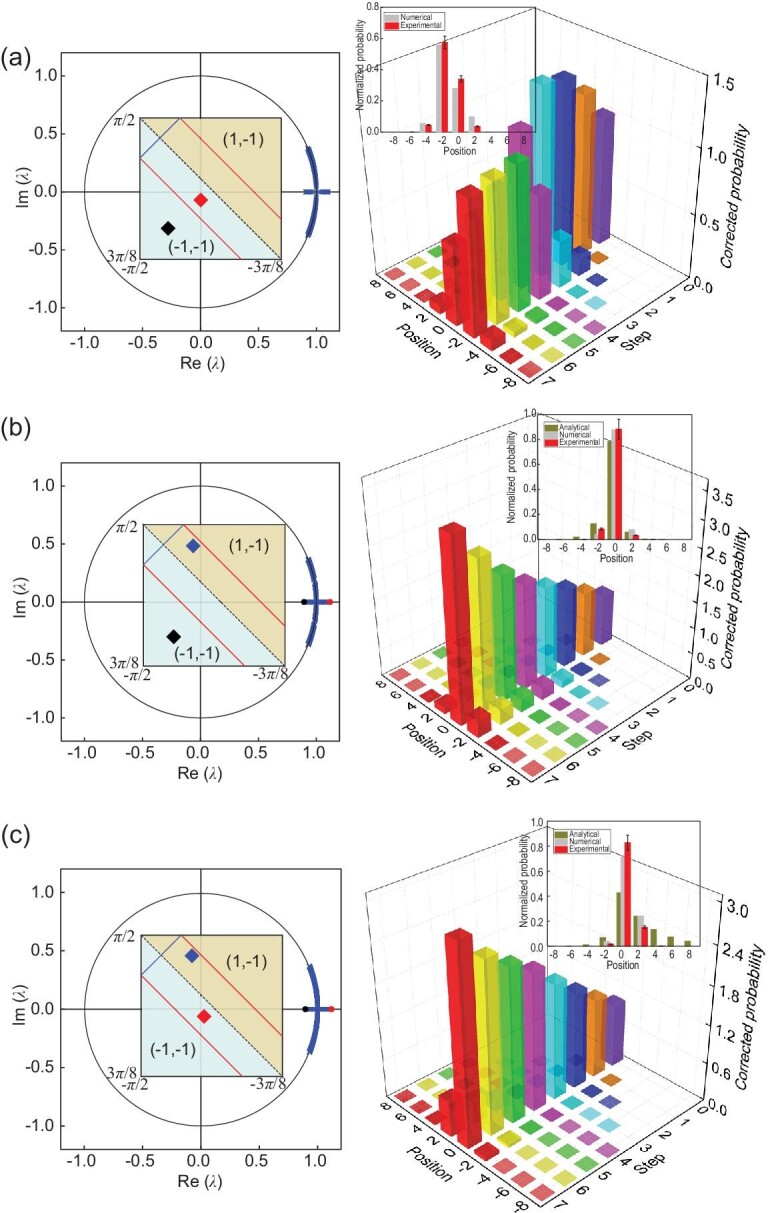
Experimental observation of topological edge states in the $\mathcal {PT}$-symmetry-broken regime. We consider QWs with fixed *p* = 9/25 and initial state $\mathinner {|{0}\rangle }\otimes \mathinner {|{+}\rangle }$. (a),(b) We fix the coin parameters $(\theta ^{\rm R}_1,\theta ^{\rm R}_2)=(-7\pi /16-\xi ,7\pi /16-\xi )$ in the right region, and vary those in the left region: $(\theta ^{\text{L}}_1,\theta _2^{\text{L}})=(-7\pi /16-\xi /4,7\pi /16-\xi /4)$ (a) and ( − 15π/32 + 3ξ/8, 15π/32 + 3ξ/8) (b). (c) We set $(\theta ^{\rm R}_1,\theta ^{\rm R}_2)=(-7\pi /16-\xi /4,7\pi /16-\xi /4)$ and $(\theta ^{\text{L}}_1,\theta _2^{\text{L}})=(-15\pi /32+3\xi /8,15\pi /32+3\xi /8)$. We fix ξ = 0.1113 here. Left column: eigenvalues λ in the complex plane. Right column: measured corrected probability distributions up to seven steps. Inset: comparison between the measured, numerically calculated and analytically calculated normalized probability distributions at the seventh step.

We then study topological edge states when at least one of the bulks is $\mathcal {PT}$-symmetry broken. For the first case in Fig. 3(a), both regions belong to the same topological phase with (ν_0_, ν_π_) = ( − 1, −1), whereas the left region is $\mathcal {PT}$-symmetry broken. The measured corrected probability near the boundary *x* = 0 is not enhanced, and after several steps of evolution the probability is no longer localized at the boundary.

In the second case shown in Fig. 3(b), the right region is still in the $\mathcal {PT}$-symmetry-unbroken regime with (ν_0_, ν_π_) = ( − 1, −1), whereas the left region is $\mathcal {PT}$-symmetry broken and has (ν_0_, ν_π_) = (1, −1). In the central column, our experimental results clearly show the enhancement of the corrected probability near *x* = 0, which gets amplified in time. In the right column, the measured normalized spatial probability distribution after the seventh step agrees reasonably well with the probability given by analytical edge-state wave functions. These observations confirm the existence of topological edge states in the presence of $\mathcal {PT}$-symmetry broken bulks, which indicates the robustness of topological phenomena against spontaneous $\mathcal {PT}$-symmetry breaking.

In the last case shown in Fig. 3(c), the left and right regions belong to FTPs with different topological numbers (ν_0_, ν_π_) = ( − 1, −1) and (1, −1), respectively, and both regions are $\mathcal {PT}$-symmetry broken. In the central column, our experimental results clearly show the enhancement of the corrected probability near *x* = 0, which increases with time. The measured normalized probability distribution after the seventh step, however, is not fully converged to the probability given by the analytical edge-state wave function. This suggests that it takes more time steps for the QW dynamics to converge into topological edge states in the presence of $\mathcal {PT}$-symmetry-broken bulks. Nevertheless, similar to the second case above, our results confirm the existence of topological edge states in the presence of $\mathcal {PT}$-symmetry broken bulks.

Whereas experimental signals of bright edge states are probed from enhanced local probabilities at the boundary, local signals of dark edge states are easily overwhelmed by those of the bulk states, especially when the bulk is in the $\mathcal {PT}$-symmetry-broken regime. We overcome the challenge and experimentally probe the elusive dark edge states by measuring the integrated probability *P*_I_(*t*) at each time step, where


(4) \begin{eqnarray*} P_{\rm I}(t)=\sum _x P_{\rm C}(x,t). \end{eqnarray*}

Apparently, *P*_I_(*t*) should be close to unity when quasienergies of a QW are real, which provides the basis for our detection.

**Figure 4.**
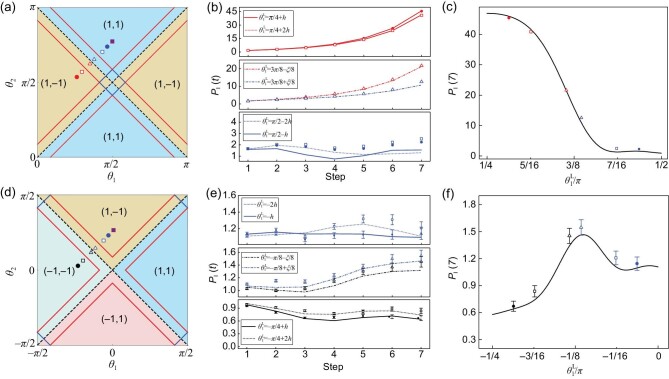
Bright and dark edge states in $\mathcal {PT}$-symmetric QWs. (a)–(c) For the bright edge states, we choose the initial state $\mathinner {|{0}\rangle }\otimes \mathinner {|{-}\rangle }$ and fix *p* = 2/3 as well as $(\theta ^{\rm R}_1,\theta ^{\rm R}_2)=(\pi /2,3\pi /4)$, and vary coin parameters for the left region along the line $\theta ^{\rm L}_2=\theta ^{\rm L}_1+\pi /4$. In the upper panel of (b), we take $\theta ^{\rm L}_1=\lbrace \pi /4+h,\pi /4+2h\rbrace$, corresponding to $\mathcal {PT}$-symmetry-unbroken regimes with different topological numbers compared to the right region. In the lower panel, $\theta ^{\rm L}_1=\lbrace \pi /2-2h,\pi /2-h\rbrace$, corresponding to $\mathcal {PT}$-symmetry-unbroken regimes with the same topological numbers compared to the right region. In the central panel, $\theta ^{\rm L}_1=\lbrace 3\pi /8-\xi/8,3\pi /8+\xi/8\rbrace$, corresponding to $\mathcal {PT}$-symmetry-broken regimes with either the same or different topological numbers compared to the right region. Here ξ′ = 0.2715 and *h* = 0.1. (d)–(f) For the dark edge states, we keep the same *p* and the line in the coin-parameter space for the left region, and we fix $(\theta ^{\rm R}_1,\theta ^{\text{R}}_2)=(0,\pi /4)$. In the upper panel of (e), $\theta ^{\rm L}_1=\lbrace -2h,-h\rbrace$, corresponding to the $\mathcal {PT}$-symmetry-unbroken regimes with the same topological numbers compared to the right region. In the lower panel, $\theta ^{\rm L}_1=\lbrace -\pi /4+h,-\pi /4+2h\rbrace$, corresponding to $\mathcal {PT}$-symmetry-unbroken regimes with different topological numbers compared to the right region. In the central panel, $\theta ^{\rm L}_1=\lbrace -\pi /8-\xi/8,-\pi /8+\xi/8\rbrace$, corresponding to $\mathcal {PT}$-symmetry-broken regimes with either the same or different topological numbers compared to the right region. Left column: the integrated probability versus the number of steps. Centre column: the integrated probability at the seventh step versus the coin parameter $\theta ^{\rm L}_1$. Right column: phase diagram, with symbols indicating the coin parameters and the corresponding topological numbers for each experimental case. Error bars in (a)–(d) are smaller than symbols and therefore not shown.

To illustrate our detection scheme, we first examine the simpler case of bright edge states. The choices of coin parameters are shown in Fig. 4(a). When the left and right regions are in the $\mathcal {PT}$-symmetry-unbroken regime with the same topological numbers [bottom panel in Fig. 4(b)], *P*_I_(*t*) remains of the order of unity at all steps. In contrast, when the two regions are in the $\mathcal {PT}$-symmetry-unbroken regime with different topological numbers [top panel in Fig. 4(b)], *P*_I_(*t*) increases monotonically over time, indicating the emergence of bright edge states. When the left region is in the $\mathcal {PT}$-symmetry-broken regime [central panel in Fig. 4(b)], *P*_I_(*t*) grows over time, even when both regions belong to the same topological phase. This is due to the existence of bulk states with complex quasienergies, which feature exponentially growing probabilities in time. However, the rate of growth in *P*_I_(*t*) is apparently larger when the two regions are in different topological phases, which is a direct result of the edge-state quasienergy having larger imaginary components. Thus, we can identify the existence of topological edge states even in the $\mathcal {PT}$-symmetry-broken regime by comparing rates of the probability growth. In Fig. 4(c), we plot *P*_I_(*t* = 7) as $\theta _1^{{\rm L}}$ is varied. The approximate location of the topological phase transition is identified as an increase of the integrated probability. Note that, due to the $\mathcal {PT}$-symmetry-broken regime around the topological phase boundary, such an increase occurs smoothly.

We now turn to dark edge states with the parameters in Fig. 4(d). In the bottom panel of Fig. 4(e), we clearly identify the existence of dark edge states as a decay of *P*_I_(*t*) over time, when the left and right regions are in the $\mathcal {PT}$-symmetry-unbroken regime with different winding numbers. This is in contrast to the central panel of Fig. 4(e), where the left region becomes $\mathcal {PT}$-symmetry broken. There *P*_I_(*t*) becomes increasing in the long-time limit, due to the emergence of bulk states with complex quasienergies. Nevertheless, *P*_I_(*t*) increases slower in the presence of dark edge states, which is the result of edge-state quasienergy having larger imaginary components and hence larger decay rate. In Fig. 4(f), we plot *P*_I_(*t* = 7) as a function of $\theta _1^{\text{L}}$, where the location of the topological phase transition is approximately identified as a decrease in the integrated probability.

### Robustness of edge states against disorder

A key feature of topologically non-trivial systems is the robustness of topological properties against small perturbations. We experimentally confirm the robustness of the topological edge states by introducing static disorder to the coin rotations. The static disorder breaks $\mathcal {PT}$ symmetry, but preserves the pseudo-anti-unitarity of $\tilde{U}^{\prime }$.

**Figure 5.**
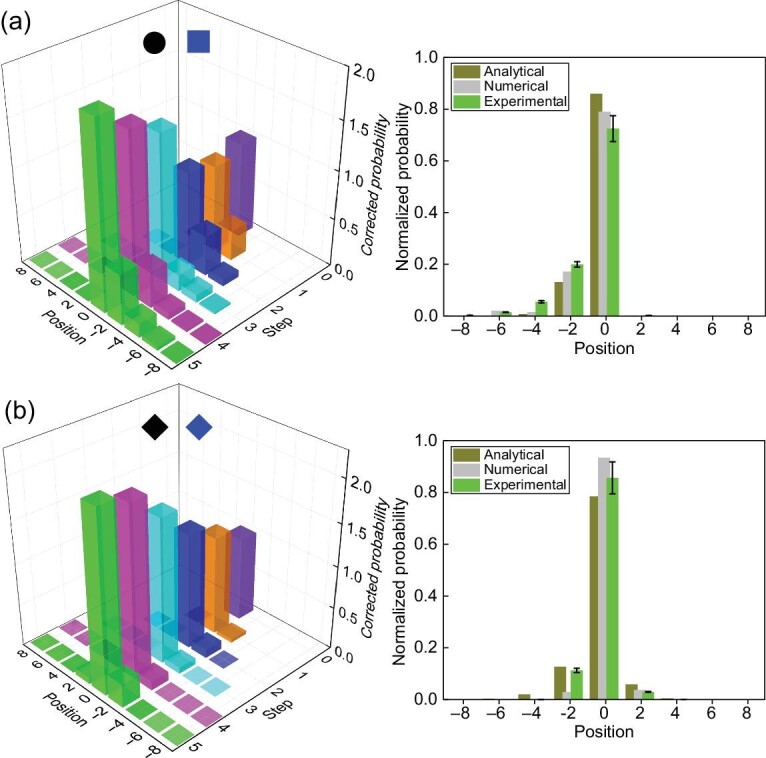
Robustness of edge states against static disorder. Probability distributions of five-step QWs with initial state $\mathinner {|{0}\rangle }\otimes \mathinner {|{+}\rangle }$ and parameter *p* = 9/25. (a) The coin parameters are the same as those in Fig. 2(b): $(\langle \theta ^{\text{R}}_1\rangle ,\langle \theta ^{\text{R}}_2\rangle )=(-9\pi /16,-5\pi /16)$ and $(\langle \theta ^{\text{L}}_1\rangle ,\langle \theta ^{\text{L}}_2\rangle )=(\pi /16,5\pi /16)$ (ξ = 0.1113). (b) The coin parameters are the same as those in Fig. 3(b): $(\langle \theta ^{\text{R}}_1\rangle ,\langle \theta ^{\text{R}}_2\rangle )=(-7\pi /16-\xi ,7\pi /16-\xi )$ and $(\langle \theta ^{\text{L}}_1\rangle ,\langle \theta ^{\text{L}}_2\rangle )=(-15\pi /32+3\xi /8,15\pi /32+3\xi /8)$. The disordered rotation angles are given by θ_1, 2_ + δθ, where δθ is unique for each position and is independent of time and chosen from the intervals [ − ξ/4, ξ/4]. Left column: measured corrected probability up to five steps. Right column: comparison between the measured and numerically calculated normalized probability distribution at the fifth step, as well as that calculated from the analytical edge-state wave functions.

First, we study the robustness of topological edge states when both regions are in the $\mathcal {PT}$-symmetry-unbroken regime. We introduce static disorder to the coin rotations by modulating the setting angles of the corresponding HWPs by a small random amount δθ ∈ [- ξ/4, ξ/4] around $\theta ^{\rm L,R}_{1,2}$. Here δθ is time independent and unique for each position. We then measure the probabilities of the walker up to five steps. As shown in Fig. 5(a), the measured corrected probability at *x* = 0 increases with time (left), while the normalized probability after the fifth step converges to that given by the analytical edge-state wave function (right). These observations confirm the robustness of edge states in the $\mathcal {PT}$-symmetry-unbroken regime against the static disorder.

Second, we choose a $\mathcal {PT}$-symmetry-broken left region and a $\mathcal {PT}$-symmetry-unbroken right region, respectively. As shown in Fig. 5(b), the measured corrected probability demonstrates signals of localized edge states, thus confirming the robustness of topological edge states against static disorder even in the $\mathcal {PT}$-symmetry-broken regime.

In the presence of strong disorder, some of the disorder-induced localized bulk states can acquire imaginary quasienergies, which may impact the detection of topological edge states through the integrated probability *P*_I_(*t*). We perform numerical simulations in the presence of strong disorder, and demonstrate that even in this case, the existence of topological edge states can still be inferred from *P*_I_(*t*).

**Figure 6.**
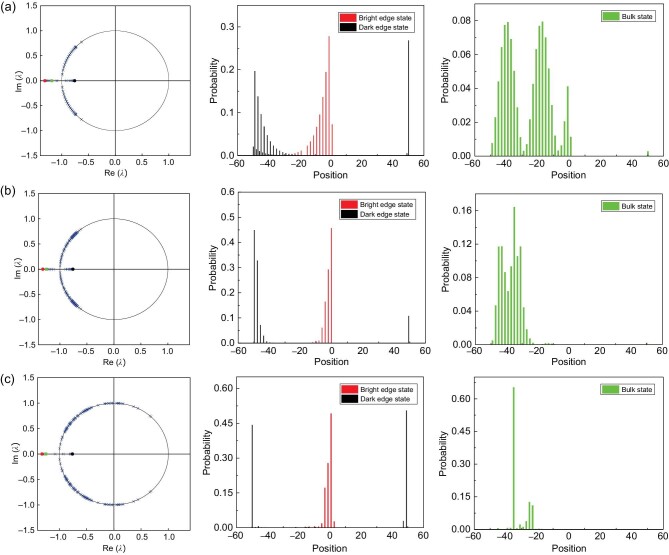
Influence of disorder on edge states and bulk states. The coin parameters are $(\theta ^{\text{R}}_1,\theta ^{\text{R}}_2)=(\pi /2,3\pi /4)$ and $(\theta ^{\text{L}}_1,\theta ^{\text{L}}_2)=(3\pi /8-\xi ^{\prime }/8,5\pi /8-\xi ^{\prime }/8)$ for the case of no disorder [red triangle in Fig. 4(a)]; $(\theta ^{\text{R}}_1,\theta ^{\text{R}}_2)=(\pi /2+\delta _1^R,3\pi /4+\delta _2^R)$, $(\theta ^{\text{L}}_1,\theta ^{\text{L}}_2)=(\delta _1^L,-\theta _1^L+\pi -\xi ^{\prime }/2+\delta _2^L)$ for the case of weak disorder and $(\theta ^{\text{R}}_1,\theta ^{\text{R}}_2)=(\pi /2+\Delta _1^R,3\pi /4+\Delta _2^R)$, $(\theta ^{\text{L}}_1,\theta ^{\text{L}}_2)=(\Delta _1^L,-\theta _1^L+\pi -\xi ^{\prime }/2+\Delta _2^L)$ for the case of strong disorder, respectively. Parameter ξ′ = 0.2715 is the same as in Fig. 4. Here $(\delta _1^R,\delta _2^R)\in [-\pi /32+\xi ^{\prime }/8,\pi /32-\xi ^{\prime }/8]$, $\delta _1^L\in [3\pi /8-\xi ^{\prime }/8,3\pi /8]$, $\delta _2^L\in [-\xi ^{\prime }/2,\xi ^{\prime }/2]$, $(\Delta _1^R,\Delta _2^R)\in [-\pi /8+\xi ^{\prime }/2,\pi /8-\xi ^{\prime }/2]$, $\Delta _1^L\in [0,\pi /2-\xi ^{\prime }]$ and $\Delta _2^L\in [-\xi ^{\prime }/2,\xi ^{\prime }/2]$ are unique for each position and are independent of time. Parameter *p* = 9/25 and ξ = 0.1113. Left column: the eigenvalue spectra λ for (a) no disorder, (b) weak disorder and (c) strong disorder on the complex plane. Central column: the spatial probability distribution of localized topological edge states. Right column: the spatial probability distribution of bulk states.

In Fig. 6, we compare the eigenspectra, and the edge-state and bulk-state wave functions with increasing strength of disorder. We find that, as the strength of disorder increases, both $\mathcal {PT}$-symmetry-unbroken and -broken bulk states become rather localized, and there are an increasing number of $\mathcal {PT}$-symmetry-broken bulk states. However, in all cases, quasienergies of topological edge states still have the largest magnitude, as indicated by red and black dots in Fig. 6. These should dictate that *P*_I_(*t*) still be dominated by contributions from the bright edge states.

**Figure 7.**
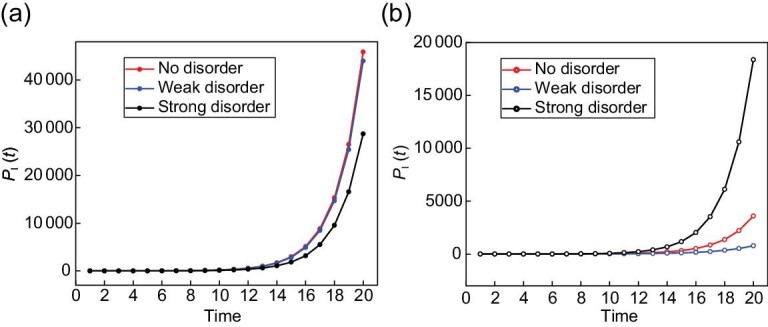
Robustness of the integrated probability against static disorder. The integrated probability *P*_I_(*t*) of 20-step QWs with initial state $\mathinner {|{0}\rangle }\otimes \mathinner {|{-}\rangle }$. (a) The coin parameters are the same as those in Fig. 6. (b) The coin parameters are $(\theta ^{\text{R}}_1,\theta ^{\text{R}}_2)=(\pi /2,3\pi /4)$ and $(\theta ^{\text{L}}_1,\theta ^{\text{L}}_2)=(3\pi /8+\xi ^{\prime }/8,5\pi /8+\xi ^{\prime }/8)$ for the case of no disorder [blue triangle in Fig. 4(a)]; $(\theta ^{\text{R}}_1,\theta ^{\text{R}}_2)=(\pi /2+\delta _1^R,3\pi /4+\delta _2^R)$, $(\theta ^{\text{L}}_1,\theta ^{\text{L}}_2)=(\delta _1^L,-\theta _1^L+\pi +\xi ^{\prime }/2+\delta _2^L)$ for the case of weak disorder and $(\theta ^{\text{R}}_1,\theta ^{\text{R}}_2)=(\pi /2+\Delta _1^R,3\pi /4+\Delta _2^R)$, $(\theta ^{\text{L}}_1,\theta ^{\text{L}}_2)=(\Delta _1^L,-\theta _1^L+\pi +\xi ^{\prime }/2+\Delta _2^L)$ for the case of strong disorder, where $(\delta _1^R,\delta _2^R)\in [-\pi /32+\xi ^{\prime }/8,\pi /32-\xi ^{\prime }/8]$, $\delta _1^L\in [3\pi /8,3\pi /8+\xi ^{\prime }/8]$, $\delta _2^L\in [-\xi ^{\prime }/2,\xi ^{\prime }/2]$, $(\Delta _1^R,\Delta _2^R)\in [-\pi /8+\xi ^{\prime }/2,\pi /8-\xi ^{\prime }/2]$, $\Delta _1^L\in [\xi ^{\prime },\pi /2]$ and $\Delta _2^L\in [-\xi ^{\prime }/2,\xi ^{\prime }/2]$. Here *p* = 9/25 and ξ′ = 0.2715.

As we show in Fig. 7, this is indeed the case. The integrated probability *P*_I_(*t*) still increases exponentially in the presence of strong disorder. Whereas the rate of increase is slower compared to cases with weaker disorder, due to the presence of $\mathcal {PT}$-symmetry-broken bulk states, the growth rate in *P*_I_(*t*) in the presence of topological edge states is still much greater than in the absence of edge states.

## DISCUSSION

By confirming the existence of topological properties in both the $\mathcal {PT}$-symmetry-unbroken and-broken regimes, our results clarify the relation between non-unitary dynamics, $\mathcal {PT}$ symmetry and topology in one-dimensional topological systems with pseudo-anti-unitarity. In particular, our topological invariants are also capable of characterizing topological properties in non-unitary dynamics without explicit $\mathcal {PT}$ symmetry [30,31,64,65], where topological numbers calculated through the global Berry phase are equivalent to generalized winding numbers associated with complex-valued pseudo-spin vectors of Bloch Hamiltonians. The topological invariant defined here thus provides a unified description for non-unitary QW dynamics either with or without explicit $\mathcal {PT}$ symmetry, thus enabling two previously separate branches of research to be understood and treated on common grounds. In light of a recent experiment where topology in the anti-$\mathcal {PT}$ regime is probed using electrical circuits [39], it would be interesting to experimentally test our description in the anti-$\mathcal {PT}$ regime, which can be reached in our quantum-walk setup by further increasing the gain-loss parameter γ. Furthermore, by probing the $\mathcal {PT}$-symmetry-broken bright and dark edge states, we reveal the interesting interplay of $\mathcal {PT}$ symmetry and topology in the non-unitary dynamics. Whereas bright and dark edge states can have useful applications in topological mode selection in various settings [36], our work is the first experimental characterization of dark edge states, which represents a significant step toward a deeper understanding of topological features in $\mathcal {PT}$-symmetric systems.

## METHODS

### The effective Hamiltonian

For completeness, we derive the explicit form of the non-Hermitian effective Hamiltonian *H*_eff_. We focus on the homogeneous case with $\theta ^{\text{L}}_{1,2}=\theta ^{\text{R}}_{1,2}=\theta _{1,2}$, which allows us to write $\tilde{U}^{\prime }$ in momentum space


(5) \begin{eqnarray*} \tilde{U}^{\prime } = d_0\mathbb {1}_c-id_1\sigma _x-id_2\sigma _y-id_3\sigma _z, \end{eqnarray*}

(6) \begin{eqnarray*} d_0 = \alpha (\cos 2k\cos \theta _1\cos \theta _2-\sin \theta _1\sin \theta _2), \end{eqnarray*}

(7) \begin{eqnarray*} d_1 = i\beta , \end{eqnarray*}

(8) \begin{eqnarray*} d_2 &= \alpha (\cos 2k\cos \theta _2\sin \theta _1+\cos \theta _1\sin \theta _2), \end{eqnarray*}

(9) \begin{eqnarray*} d_3 &=-\alpha \sin 2k\cos \theta _2, \end{eqnarray*}

(10) \begin{eqnarray*} {d^2_0 + d^2_1+d^2_2+d^2_3 = \alpha ^2-\beta ^2=1}, \end{eqnarray*}

where $\alpha =\gamma (1+\sqrt{1-p})/2 $, $\beta =\gamma (1-\sqrt{1-p})/2 $, the *d_i_* (*i* = 0, 1, 2, 3) are momentum dependent, the σ_*x, y, z*_ are the Pauli matrices and $\mathbb {1}_c $ is a two-by-two identity matrix.

The eigenvalues of $\tilde{U}^{\prime }$ are given by $\lambda _{\pm } = d_0\mp i\sqrt{1-d_0^2}$, where ± are band indices. Note that λ_+_λ_−_ = 1, which is guaranteed by $\mathcal {PT}$ symmetry of the Floquet operator $\tilde{U}^{\prime }$. As we define the effective Hamiltonian through $\tilde{U}^{\prime }=\exp (-i H_{\rm eff})$, the quasienergy spectrum of *H*_eff_ is given by ε_±_ = *i *ln (λ_±_). We first define the two bands of the quasienergy spectrum of *H*_eff_ as ε_±_, which are given by ε_±_ = *i *ln (λ_±_). The effective Hamiltonian is then


(11) \begin{eqnarray*} H_{\rm {eff}}=h_0\mathbb {1}_c+h_1\sigma _x+h_2\sigma _y+h_3\sigma _z, \end{eqnarray*}

where *h*_0_ = (ε_+_ + ε_−_)/2 and $h_i= {(\epsilon _+ -\epsilon _-)}d_i / {(2\sqrt{1-d_0^2})},\, i=1,2,3 $.

## Supplementary Material

nwad005_Supplemental_File
