# Supplementary material for: Observation of dark edge states in parity-time-symmetric quantum dynamics
Source: Natl Sci Rev. 2023 Jan 10;10(8):nwad005. doi: 10.1093/nsr/nwad005 (PMC10306368; doi:10.1093/nsr/nwad005)
Supplement: nwad005_Supplemental_File [file nwad005_supplemental_file.pdf]

SUPPLEMENTAL MATERIALS FOR "OBSERVATION OF DARK EDGE STATES IN A PARITY-TIME-SYMMETRIC QUANTUM DYNAMICS"

Experimental implementation of $\tilde{U}'$

With a single-photon source consisting of a β-barium-borate (BBO) nonlinear crystal pumped by a CW diode laser, we generate polarization-degenerate photon pairs at 801.6nm using a type-I spontaneous parametric down-conversion (SPDC) process. Upon detection of a trigger photon, the signal photon is heralded in the measurement setup. This trigger-signal photon pair is registered by a coincidence count at two APDs with a $\Delta t = 3$ns time window. Total coincidence counts are about $10,000$ over a collection time of 2s.

The coin states $|0\rangle$ and $|1\rangle$ are respectively encoded in the horizontal $|H\rangle$ and vertical $|V\rangle$ polarizations of the heralded single photon, whose spatial modes represent the walker state. After passing through a polarizing beam splitter (PBS) followed by a HWP, the heralded single photon is projected into an arbitrary initial state and then proceeds through the quantum-walk interferometric network. We implement the coin operator $R(\theta)$ by HWPs with certain setting angles depending on the coin parameters (θ_1, θ_2), and the shift operator S by a BD whose optical axis is cut so that the photons in $|V\rangle$ are directly transmitted and those in $|H\rangle$ undergo a lateral displacement into a neighboring spatial mode, respectively. The loss operator M is implemented by a sandwich-type HWP (at 22.5°)-PPBS-HWP (at 22.5°) setup [4]. Here, the transmissivities of PPBS are $(T_\text{H}, T_\text{V}) = (1, 1-p)$ for horizontally and vertically polarized photons, respectively.

We construct the raw probability distribution of the walker P_R at time t by dividing the number of coincidence measurements at APDs using the total number of photon pairs, after correcting for the relative efficiencies of different APDs. The raw probability is then converted into the corrected probability $P_\text{C}(x,t) = \gamma^{2t} P_\text{R}(x,t)$, which is obtained by multiplying the correction factor γ for the corresponding step t and represents the probability corresponding to parity-time ($\mathcal{PT}$)-symmetric quantum walks (QWs) governed by $\tilde{U}'$. Whereas, the normalized probability $P_\text{N}(x,t)$ is defined as $P_\text{R}(x,t)/\sum_x P_\text{R}(x,t)$.

$\mathcal{PT}$ symmetry of non-unitary quantum walks governed by $\tilde{U}'$

In this section, we discuss $\mathcal{PT}$ symmetry of two-step QWs governed by the non-unitary Floquet operator $\tilde{U}' = F\tilde{M}G$, where

$$F = R\left(\frac{\theta_1}{2}\right) SR\left(\frac{\theta_2}{2}\right), G = R\left(\frac{\theta_2}{2}\right) SR\left(\frac{\theta_1}{2}\right). \tag{S1}$$

The operators $\tilde{M}$, R, and S are defined in the main text. We focus on the homogeneous case with $\theta^\text{L}_{1,2} = \theta^\text{R}_{1,2} = \theta_{1,2}$, which allows us to write $\tilde{U}'$ in momentum space

$$\tilde{U}' = d_0 \mathbb{1}_c - id_1\sigma_x - id_2\sigma_y - id_3\sigma_z, \tag{S2}$$
$$d_0 = \alpha\left(\cos 2k \cos\theta_1 \cos\theta_2 - \sin\theta_1 \sin\theta_2\right), \tag{S3}$$
$$d_1 = i\beta, \tag{S4}$$
$$d_2 = \alpha\left(\cos 2k \cos\theta_2 \sin\theta_1 + \cos\theta_1 \sin\theta_2\right), \tag{S5}$$
$$d_3 = -\alpha \sin 2k \cos\theta_2, \tag{S6}$$
$$d_0^2 + d_1^2 + d_2^2 + d_3^2 = \alpha^2 - \beta^2 = 1, \tag{S7}$$

where $\alpha = \gamma(1+\sqrt{1-p})/2$, $\beta = \gamma(1-\sqrt{1-p})/2$ and d_i $(i = 0,1,2,3)$ are momentum dependent, $\sigma_{x,y,z}$ are the Pauli matrices, and $\mathbb{1}_c$ is a two-by-two identity matrix.

The eigenvalues of $\tilde{U}'$ are given by $\lambda_\pm = d_0 \mp i\sqrt{1-d_0^2}$, where $\pm$ are band indices. Note that $\lambda_+\lambda_- = 1$, which is guaranteed by $\mathcal{PT}$ symmetry of the Floquet operator $\tilde{U}'$. As we define the effective Hamiltonian through $\tilde{U}' = \exp(-iH_\text{eff})$, the quasienergy spectrum of H_eff is given by $\epsilon_\pm = i\ln(\lambda_\pm)$. Apparently, when $d_0^2 < 1$ for all k, the quasienergy spectrum is entirely real. In this case, the system is in the $\mathcal{PT}$-symmetry-unbroken regime. In contrast, when $d_0^2 > 1$ is satisfied for a certain range of momenta k, the corresponding quasienergies in that range become complex. In this case, the system is in the $\mathcal{PT}$-symmetry-broken regime. The transition between the above two scenarios, the so-called exceptional point, occurs when $d_0^2 = 1$ is satisfied at some discrete momenta while $d_0^2 < 1$

otherwise. At these momenta, the quasienergy band gap closes at $\epsilon = 0$ (with $\epsilon_+ = \epsilon_- = 0$) or $\epsilon = \pi$ (with $\epsilon_+ = \epsilon_- = \pi$).

We further divide the $\mathcal{PT}$-symmetry-broken regime into the partially-broken and the completely-broken regimes, where complex quasienergies occur for part of or the whole first Brillioun zone, respectively. The important difference between the $\mathcal{PT}$-symmetry-partially-broken and completely-broken cases is that the latter does not have quasienergy band gap closing, i.e., ($\epsilon_\pm \neq 0, \pi$) for any k in the symmetry completely-broken regime. In the left two columns of Fig. S1, we plot quasienergies $\epsilon_\pm$ and eigenvalues $\lambda_\pm$ for the different scenarios above.

We experimentally confirm $\mathcal{PT}$ symmetry of $\tilde{U}'$ by analyzing homogeneous QWs for both the $\mathcal{PT}$-unbroken and broken states. We start with a homogeneous QW in $\mathcal{PT}$-symmetry-unbroken regime, with the coin parameters $(\theta_1^{\mathrm{L,R}}, \theta_2^{\mathrm{L,R}}) = (-\pi/4, 3\pi/4 - 3\xi)$. We fix the parameter $\xi = 0.1113$ in our experiment. As illustrated in Fig. S1(a), all quasienergies are real and gaps are open for all momenta. Correspondingly, eigenvalues of $\tilde{U}'$ all lie on a unit circle in the complex plane. The measured corrected probability distribution is ballistic, which agrees well with numerical simulations and is similar to that of a standard unitary QW.

We then change the coin parameters to $(\theta_1, \theta_2) = (-4\pi/9, 5\pi/9 + \xi)$. The resulting QW is at the exceptional point. As illustrated in Fig. S1(b), in this case all the quasienergies are still real, but the quasienergy gap closes at $\epsilon = 0$. The measured corrected probability distribution is different from that of the standard unitary QW with a squeezed profile.

When we change the coin parameters to $(\theta_1, \theta_2) = (-17\pi/36, 19\pi/36 + \xi/2)$, the system enters the $\mathcal{PT}$-symmetry-partially-broken regime. As illustrated in Fig. S1(c), the quasienergies become complex. Meanwhile, some eignevalues $\lambda_\pm$ deviate from the unit circle with $\lambda_\pm > 0$, which corresponds to $\mathrm{Re}(\epsilon_\pm) = 0$. The corrected probability distribution is Gaussian-like, which is completely different from that in the $\mathcal{PT}$-symmetry-unbroken regime.

When we change the coin parameters to $(\theta_1, \theta_2) = (-\pi/2 - 3\xi/8, -\pi/2 - 3\xi/8)$, the system enters the $\mathcal{PT}$-symmetry-completely-broken regime. As illustrated in Fig. S1(d), all quasienergies become imaginary with $\mathrm{Re}(\epsilon_\pm) = 0$, while the quasienergy spectrum is fully gapped as $\epsilon_\pm \neq 0, \pi$. Meanwhile, all eignevalues $\lambda_\pm$ deviate from the unit circle and lie on the real axis with $\lambda_\pm > 0$, which corresponds to $\mathrm{Re}(\epsilon_\pm) = 0$. Again, the corrected probability distribution is Gaussian-like.

Global Berry phase of $\mathcal{PT}$-symmetric QWs

We discuss the definition of topological invariants for $\mathcal{PT}$-symmetric non-unitary QWs $\tilde{U}'$. For the convenience of calculation, we apply a unitary transformation to $\tilde{U}'$

$$W' = V\tilde{U}'V^\dagger = d_0 \mathbb{1}_c - i(-d_3)\sigma_x - i d_2 \sigma_y - i d_1 \sigma_z, \tag{S8}$$

where $V = e^{i\frac{\pi/2}{2}\sigma_y}$, $\sigma_{x,y,z}$ are the Pauli matrices, $\mathbb{1}_c$ is a two-by-two identity matrix, and the explicit expressions of d_i $(i = 0, 1, 2, 3)$ are given in the Supplemental Materials. Topological properties of $\tilde{U}'$ is not changed under the unitary transformation. We will show that the winding number ν' is defined through the global Berry phase as $\nu' = \varphi_{\mathrm{B}}/2\pi$. Here, $\varphi_{\mathrm{B}} = \varphi_{Z+} + \varphi_{Z-}$, with the generalized Zak phases for the jth band ($j = \pm$)

$$\varphi_{Z\pm} = -i \oint \mathrm{d}k \frac{\langle \chi_\pm | \frac{\mathrm{d}}{\mathrm{d}k} | \psi_\pm \rangle}{\langle \chi_\pm | \psi_\pm \rangle}. \tag{S9}$$

Here, the integral is over the first Brillioun zone and $\langle \chi_j |$ and $|\psi_j\rangle$ are respectively the left and right eigenstates of W', defined through $W'^\dagger |\chi_j\rangle = \lambda_j^* |\chi_j\rangle$ and $W' |\psi_j\rangle = \lambda_j |\psi_j\rangle$, respectively.

In the following, let us first evaluate the Berry connection

$$A_\pm = -i \frac{\langle \chi_\pm | \frac{\mathrm{d}}{\mathrm{d}k} | \psi_\pm \rangle}{\langle \chi_\pm | \psi_\pm \rangle}, \tag{S10}$$

which critically depends on whether d_0^2 is greater than 1 or not. As different momenta are decoupled, we will examine the Berry connection case by case.

Case I: the momentum region with $d_0^2 < 1$:— In the momentum regime with $d_0^2 < 1$, we have $d_1^2 + d_2^2 + d_3^2 > 0$, and the right and the left eigenvectors of W' are

$$|\psi_\pm\rangle = \frac{1}{\sqrt{2\cos 2\Omega}} (\pm e^{\pm i\Omega}, e^{+i\vartheta} e^{\mp i\Omega})^{\mathrm{T}}, \tag{S11}$$

$$\langle \chi_\pm | = \frac{1}{\sqrt{2\cos 2\Omega}} (\pm e^{\pm i\Omega}, e^{-i\vartheta} e^{\mp i\Omega}). \tag{S12}$$

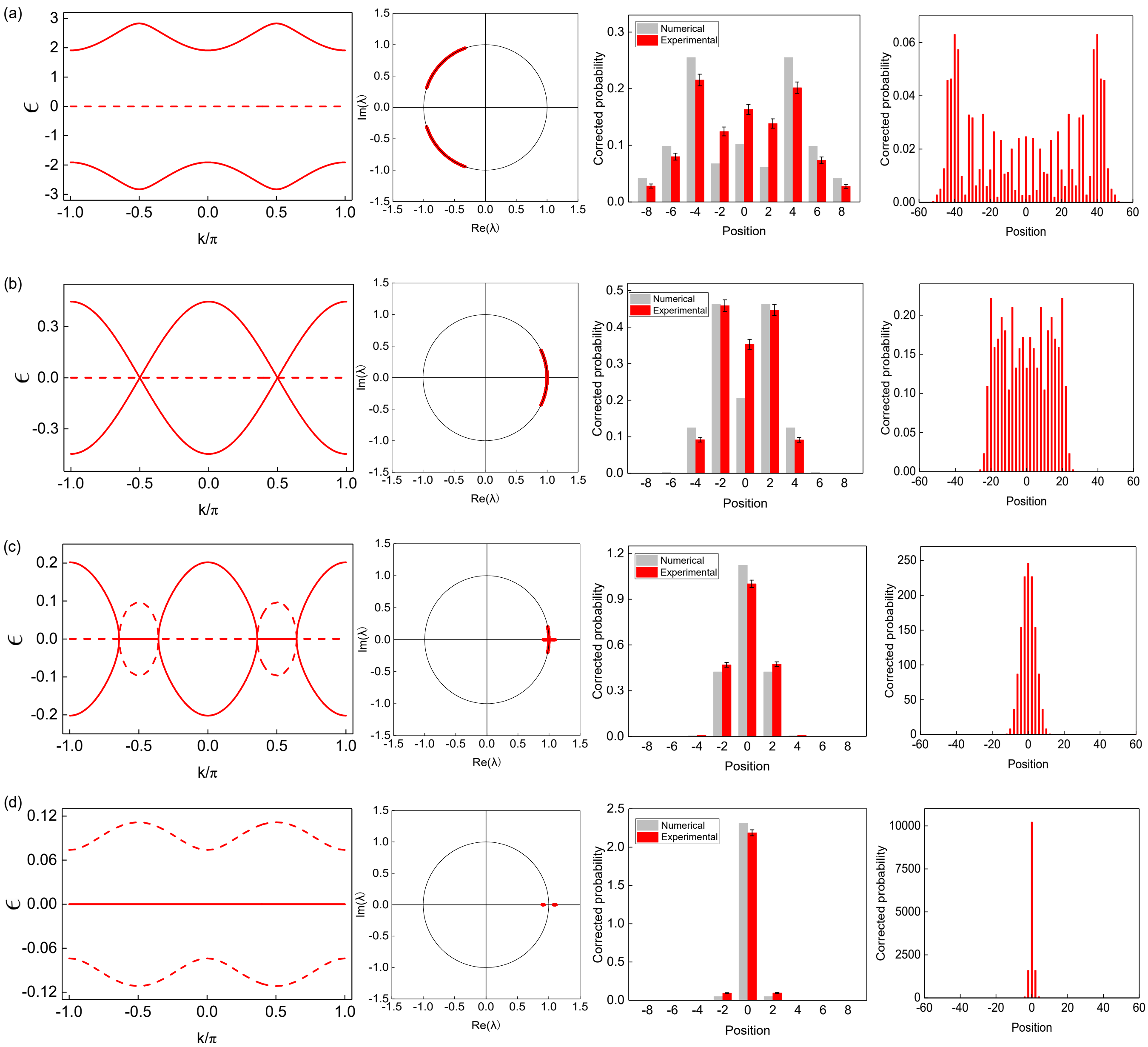

FIG. S1. **Experimental $\mathcal{PT}$-symmetric homogeneous QW with the initial state $|0\rangle \otimes (|+\rangle + i\,|-\rangle)/\sqrt{2}$.** (a) QWs with $(\theta_1, \theta_2) = (-\pi/4, 3\pi/4 - 3\xi)$ in the unbroken $\mathcal{PT}$-symmetric phase. (b) QW with $(\theta_1, \theta_2) = (-4\pi/9, 5\pi/9 + \xi)$ at the exceptional point. (c) QW with $(\theta_1, \theta_2) = (-17\pi/36, 19\pi/36 + \xi/2)$ in the partially broken $\mathcal{PT}$-symmetric phase. (d) QW with $(\theta_1, \theta_2) = (-\pi/2 - 3\xi/8, -\pi/2 - 3\xi/8)$ in the completely broken $\mathcal{PT}$-symmetric phase. The first column: the quasienergy as a function of quasimomentum where the solid (dashed) curves represent the real (imaginary) part of quasienergy. The second column: analytical results of the eigenvalues of the time-evolution operator in the complex plane. The third column: comparison between the measured (red bars) and the predicted (grey bars) probabilities after the seventh step with different coin parameters. The fourth column: the predicted probabilities after fifty steps with different coin parameters. Experimental errors are due to photon-counting statistics and represent the corresponding standard deviations.

Here ϑ and Ω are respectively defined through $-d_3 + id_2 = de^{i\vartheta}$ and $\sin 2\Omega = -id_1/d$, with $d^2 = d_2^2 + d_3^2$. As $d_1/d \in (0, 1)$, we set $2\Omega \in (0, \pi/2)$ and $\cos 2\Omega > 0$. Notice that the orthonormal conditions ($\langle\chi_\pm|\psi_\pm\rangle = 1, \langle\chi_\pm|\psi_\mp\rangle = 0$) are always satisfied in this region. We then have

$$\frac{\mathrm{d}}{\mathrm{d}k}\,|\psi_\pm\rangle = \frac{1}{(2\cos 2\Omega)^{3/2}}\Big(i2e^{\mp i\Omega}\Omega', ie^{i\vartheta}e^{\mp i\Omega}(2\cos 2\Omega)\vartheta' \mp i2e^{i\vartheta}e^{\pm i\Omega}\Omega'\Big)^{\mathrm{T}}, \tag{S13}$$

where $\Omega' = \mathrm{d}\Omega/\mathrm{d}k$ and $\vartheta' = \mathrm{d}\vartheta/\mathrm{d}k$. It is then straightforward to derive $A_\pm = \frac{1}{2}\vartheta' \pm \frac{i}{2}\vartheta' \tan 2\Omega$, and $A_+ + A_- = \vartheta'$.

Case II: the momentum region with $d_0^2 > 1$:— In the momentum regime with $d_0^2 > 1$, we have $d_1^2 + d_2^2 + d_3^2 < 0$, and the right and the left eigenvectors of W' are

$$|\psi_\pm\rangle = \frac{1}{\sqrt{\mp 2\sinh 2\Xi}}(ie^{\pm\Xi}, e^{+i\vartheta}e^{\mp\Xi})^{\mathrm{T}}, \tag{S14}$$

$$\langle\chi_\pm| = \frac{1}{\sqrt{\mp 2\sinh 2\Xi}}(ie^{\pm\Xi}, e^{-i\vartheta}e^{\mp\Xi}), \tag{S15}$$

where $\cosh 2\Xi = -id_1/d$, with $\Xi \in (0, \infty)$. We then have

$$\frac{\mathrm{d}}{\mathrm{d}k}|\psi_\pm\rangle = \frac{1}{(\mp 2\sinh 2\Xi)^{3/2}}\Big(\pm i2\Xi' e^{\mp\Xi}, \mp i2\vartheta' \sinh 2\Xi e^{i\vartheta} e^{\mp\Xi} \pm 2\Xi' e^{i\vartheta} e^{\pm\Xi}\Big)^{\mathrm{T}}, \tag{S16}$$

where $\Xi' = \mathrm{d}\Xi/\mathrm{d}k$. The Berry connection is then $A_\pm = \pm\frac{e^{\mp 2\Xi}}{2\sinh 2\Xi}\vartheta'$. Again, we have $A_+ + A_- = \vartheta'$.

Case III: at discrete momenta with $d_0^2 = 1$:— In this case, $d_1^2 + d_2^2 + d_3^2 = 0$. The right and the left eigenvectors of W' are

$$|\psi_\pm\rangle = \frac{1}{\sqrt{2}}(i, e^{+i\theta})^{\mathrm{T}}, \tag{S17}$$

$$\langle\chi_\pm| = \frac{1}{\sqrt{2}}(i, e^{-i\theta}). \tag{S18}$$

As $\langle\chi_\pm|\psi_\pm\rangle = \langle\chi_\pm|\psi_\mp\rangle = 0$ and $\langle\chi_\pm|\chi_\pm\rangle = \langle\psi_\pm|\psi_\pm\rangle = 1$, the denominator in the Berry connection $\langle\chi_\pm|\psi_\pm\rangle$ vanishes, giving rise to diverging $A_\pm$ at these momenta. However, a closer examination reveals that the divergence in A_+ and A_- cancels out in their summation $A_+ + A_-$, giving rise to a well-defined "global Berry connection". For example, if the condition $d_0^2 = 1$ is approached in parameter space from the side with $d_0^2 < 1$, we have $A_\pm = \frac{1}{2}\vartheta' \pm \frac{i}{2}\vartheta' \tan 2\Omega$. At $d_0^2 = 1$, $\tan 2\Omega \to \infty$. The imaginary parts of Berry connections $A_\pm$ diverge, however, their summation is still ϑ' and remains well-defined even at $d_0^2 = 1$. The situation is similar when the condition $d_0^2 = 1$ is approached in parameter space from the side with $d_0^2 > 1$, where $A_\pm$ diverge but their sum is not. With the above analysis, we see that the global Berry phase is given by $\varphi_{\mathrm{B}} = \oint \mathrm{d}\vartheta$, regardless of whether the system is $\mathcal{PT}$-symmetry unbroken or broken.

In contrast, at the topological phase boundary, the polar angle ϑ in the above expressions become ill-defined as $d_2 = d_3 = 0$. This occurs at $k = 0, \pi$ for $d_0(k) = \alpha$, or at $k = \pm\pi/2$ for $d_0(k) = -\alpha$. As such, the global Berry phase can no longer be defined on the topological phase boundary. This conclusion is the same as the Zak phase of a unitary QW.

In previous studies, both generalized Zak phases and generalized winding numbers have been proposed to serve as topological invariants for non-unitary FTPs. The generalized Zak phase, which is only valid in the $\mathcal{PT}$-symmetry-unbroken regime, can be written as $\mathrm{Re}\,(\varphi_{Z-})$. As $\varphi_{Z-} = \varphi_{Z+}^*$ in the $\mathcal{PT}$-symmetry-unbroken regime, the generalized Zak phase defined in Refs. [1, 2] is equivalent to the global Berry phase φ_{B} in the $\mathcal{PT}$-symmetry-unbroken regime.

On the other hand, according to Refs. [3, 4], the generalized winding number for the Floquet operator $\tilde{U}'$ is defined as

$$\nu_1 = \frac{1}{2\pi}\oint \mathrm{d}k\left(\boldsymbol{n} \times \frac{\partial \boldsymbol{n}}{\partial k}\right)_x, \tag{S19}$$

where the unit vector $\boldsymbol{n}$ is a normalized projection of the vector $\boldsymbol{d} = (d_1, d_2, d_3)^{\mathrm{T}}$ in the y-z plane. As such, $\boldsymbol{n} = (0, d_2/d, d_3/d)^{\mathrm{T}}$. It is then straightforward to show that $\vartheta' = (\boldsymbol{n} \times \frac{\partial \boldsymbol{n}}{\partial k})_x$, such that the generalized winding number is $\nu_1 = \varphi_{\mathrm{B}}/2\pi = \nu'$. Topological invariants defined through the global Berry phase thus unify previous definitions in different contexts.

By fitting the Floquet operator $\tilde{U}'$ in a different time frame

$$\tilde{U}'' = R\left(\frac{\theta_2}{2}\right) SR\left(\frac{\theta_1}{2}\right) \tilde{M} R\left(\frac{\theta_1}{2}\right) SR\left(\frac{\theta_2}{2}\right), \tag{S20}$$

we define another winding number ν'' through the global Berry phase of $\tilde{U}''$ [1, 5]. We then construct the topological numbers $(\nu_0, \nu_\pi) = (\frac{\nu' - \nu''}{2}, \frac{\nu' + \nu''}{2})$. In the following section, we will confirm through numerical calculations that the topological numbers (ν_0, ν_π) are directly related to the number of topological edge states at a given interface. Specifically, the number of edge states with quasienergy $\mathrm{Re}(\epsilon) = 0$ [$\mathrm{Re}(\epsilon) = \pi$] is equal to the difference of topological numbers ν_0 (ν_π) on either side of the boundary.

Topological number and topological edge states

In this section, we numerically confirm that localized topological edge states at a given boundary are dictated by the difference in topological numbers (ν_0, ν_π) of the bulks on either side. More specifically, topological number ν_0 (ν_π) is associated with the number of topological edge states with $\mathrm{Re}(\epsilon) = 0$ [$\mathrm{Re}(\epsilon) = \pi$]. For convenience, we define $\Delta\nu_g = |\nu_g^{\mathrm{L}} - \nu_g^{\mathrm{R}}|$ ($g = 0, \pi$), where ν_g^{L} (ν_g^{R}) is the topological number in the left (right) region.

We numerically diagonalize Floquet operators of inhomogeneous QWs governed by $\tilde{U}'$ with $N = 50$. As illustrated in Fig. S2(a), when the difference in topological numbers is $(\Delta\nu_0, \Delta\nu_\pi) = (2, 0)$, a pair of degenerate edge states, on odd and even sites respectively, exist at a given boundary ($x = 0$ or $x = 50$) with $\mathrm{Re}(\epsilon) = 0$ [$\mathrm{Re}(\lambda) > 0$]. We note that both regions belong to the $\mathcal{PT}$-symmetry-unbroken regime. In this case, bright edge states with $\lambda > 1$ (red) appear near $x = 0$, while dark edge states with $\lambda < 1$ (black) appear near $x = 50$. In contrast, for bulk states with $|\lambda| = 1$, their spatial distributions are extended (green and orange).

In Fig. S2(b), both regions are in the $\mathcal{PT}$-symmetry-unbroken regime, and the difference in topological numbers is $(\Delta\nu_0, \Delta\nu_\pi) = (0, 2)$. A pair of degenerate edge states, on odd and even sites respectively, exist at a given boundary with $\mathrm{Re}(\epsilon) = \pi$ [$\mathrm{Re}(\lambda) < 0$]. In this case, bright edge states with $\lambda < -1$ (red) appear near $x = 0$, while dark edge states with $\lambda > -1$ (black) appear near $x = 50$. In contrast, for bulk states with $|\lambda| = 1$, their spatial distributions are extended (green and orange).

In Fig. S2(c), we consider the case where both left and right regions are in the $\mathcal{PT}$-symmetry-broken regime. The difference in topological numbers is $(\Delta\nu_0, \Delta\nu_\pi) = (2, 0)$. In this case, while $\mathcal{PT}$-symmetry-broken bulk states exist with $|\lambda| \neq 1$, localized edge states can still be identified as their eigenvalues λ deviate most from the unit circle. In terms of quasienergy, topological edge states in this case possess quasienergies with the largest imaginary parts. As shown in the central and right columns, localized edge states and extended bulk states are also differentiated by their distinct spatial probability distributions.

To summarize, we have numerically confirmed that the number of localized edge states is governed by $\Delta\nu_0$ and $\Delta\nu_\pi$. We have further checked (not shown) that such a relation holds when both topological numbers are different. Such a bulk-boundary correspondence exists even when one or both of the bulks are in the $\mathcal{PT}$-symmetry-broken regime. For all the cases shown in Fig. S2, bright (dark) edge states exist near $x = 0$ ($x = 50$). We note that their spatial location would be switched when we exchange the coin parameters of the left and right regions.

Edge-state wave functions

In this section, we solve for the wave function of topological edge states in $\mathcal{PT}$-symmetry non-unitary QWs governed by $\tilde{U}'$. We first consider the unitary QW with $p = 0$ ($\gamma = 1$), and derive the analytical solution of topological edge states near the boundary $x = 0$. We then demonstrate that topological edge states in the non-unitary case ($\gamma \neq 1$) have the same eigen wave functions as the unitary case. The difference lies in eigenvalues and hence the time evolution, where topological edge states in the non-unitary case acquire factors $\gamma^{\pm t}$, giving rise to the bright and dark edge states as discussed in the main text.

Edge states in the unitary case

In the unitary case, we write the Floquet operator as $\bar{U}' = FG$, where F and G are defined in the main text. In the homogeneous case with $\theta_{1,2}^{\mathrm{L}} = \theta_{1,2}^{\mathrm{R}} = \theta_{1,2}$, eigenvalues and eigenstates of $\bar{U}'$ are given in momentum space as

$$\begin{aligned} \bar{\lambda}_+ &= e^{-iE}, \;\; |\bar{\psi}_+\rangle = \begin{bmatrix} -\cos\theta_2 \sin 2k + \sin E \\ i(\cos\theta_2 \sin\theta_1 \cos 2k + \cos\theta_1 \sin\theta_2) \end{bmatrix}, \\ \bar{\lambda}_- &= e^{iE}, \;\; |\bar{\psi}_-\rangle = \begin{bmatrix} -\cos\theta_2 \sin 2k - \sin E \\ i(\cos\theta_2 \sin\theta_1 \cos 2k + \cos\theta_1 \sin\theta_2) \end{bmatrix}, \end{aligned} \tag{S21}$$

where $\bar{U}'|\bar{\psi}_\pm\rangle = \bar{\lambda}_\pm|\bar{\psi}_\pm\rangle$, and the quasienergy E satisfies $\cos E = \cos\theta_1 \cos\theta_2 \cos 2k - \sin\theta_1 \sin\theta_2$.

In the homogeneous case, eigenstates of $\bar{U}'$ at a given momentum k can be written as

$$|\bar{\psi}_\pm(x)\rangle = e^{ikx}\,|\bar{\psi}_\pm\rangle\,. \tag{S22}$$

In the inhomogeneous case with $\theta_{1,2}^{\mathrm{L}} \neq \theta_{1,2}^{\mathrm{R}}$, topological edge states with quasienergies $E^{(0)} = 0$ or $E^{(\pi)} = \pi$ emerge near boundaries between different bulk topological phases. Wave functions of topological edge states near

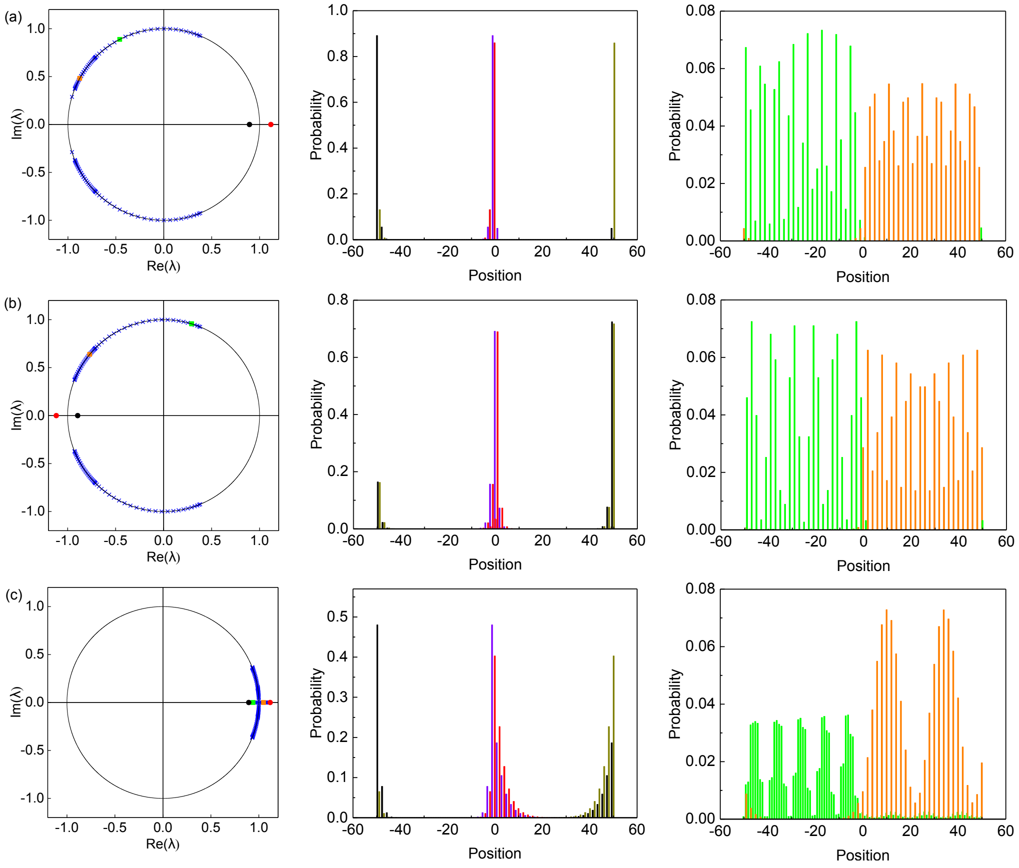

FIG. S2. **Numerical evidence for the bulk-boundary correspondence.** We numerically diagonalize Floquet operators for inhomogeneous QWs and examine the relation between the difference in bulk topological numbers and the number of localized edge states. The left column demonstrates the eigenvalue spectra λ for different cases on the complex plane. The central column shows the spatial probability distribution of localized topological edge states. The right column shows the spatial probability distribution of extended bulk states. (a) Coin parameters are the same as those in Fig. 2(b) of the main text. (b) Coin parameters are $(\theta_1^{\mathrm{L}}, \theta_2^{\mathrm{L}}) = (\pi/16, 5\pi/16)$, $(\theta_1^{\mathrm{R}}, \theta_2^{\mathrm{R}}) = (7\pi/16, 11\pi/16)$. (c) Coin parameters are the same as those in Fig. 3(c) of the main text.

the boundary $x = 0$ can be constructed from Eq. (S22) by setting $k = -i\kappa_{\mathrm{L}}$ and $k = i\kappa_{\mathrm{R}}$ for the left and right regions, respectively. Note $\mathrm{Re}\,(\kappa_{\mathrm{L}})\,, \mathrm{Re}\,(\kappa_{\mathrm{R}}) > 0$ so that probability distributions of the localized edge states vanish as $|x| \to \infty$. We further notice that under the two-step QW $\bar{U}'$, wave functions on even sites and odd sites are decoupled.

The considerations above enable us to construct wave functions for topological edge states at the boundary near $x = 0$

$$|\psi^{\mathrm{o(e)}}(x)\rangle = \begin{cases} r^{\mathrm{o(e)}} e^{\kappa_{\mathrm{L}} x} (a^{\mathrm{L}}_{-i\kappa_{\mathrm{L}}}, b^{\mathrm{L}}_{-i\kappa_{\mathrm{L}}})^{\mathrm{T}}, & x < 0, \\ t^{\mathrm{o(e)}} e^{-\kappa_{\mathrm{R}} x} (a^{\mathrm{R}}_{i\kappa_{\mathrm{R}}}, b^{\mathrm{R}}_{i\kappa_{\mathrm{R}}})^{\mathrm{T}}, & x \geq 0, \end{cases} \tag{S23}$$

where $|\psi^{\mathrm{o(e)}}(x)\rangle$ is the edge-state wave function on odd (even) sites, and $r^{\mathrm{o(e)}}$ and $t^{\mathrm{o(e)}}$ are the corresponding coefficients. We also have $\begin{bmatrix} a_k^\xi \\ b_k^\xi \end{bmatrix} = \begin{bmatrix} -\cos\theta_2^\xi \sin 2k \\ i(\cos\theta_2^\xi \sin\theta_1^\xi \cos 2k + \cos\theta_1^\xi \sin\theta_2^\xi) \end{bmatrix}$ $(\xi = \mathrm{L}, \mathrm{R})$, which, according to Eq. (S21), denotes coin states of edge-state wave functions with $E^{(0)}$ or $E^{(\pi)}$. We will show in the following that all the coefficients above have analytical forms.

From the dispersion relations, we first establish expressions for the spatial decay rates κ_ξ $(\xi = \mathrm{L}, \mathrm{R})$

$$\cosh 2\kappa_\xi = \frac{\cos E^{(0,\pi)} + \sin\theta_1^\xi \sin\theta_2^\xi}{\cos\theta_1^\xi \cos\theta_2^\xi}. \tag{S24}$$

From Eq. (S24), it is immediately clear that spatial decay rates to the left (right) of the boundary are determined by

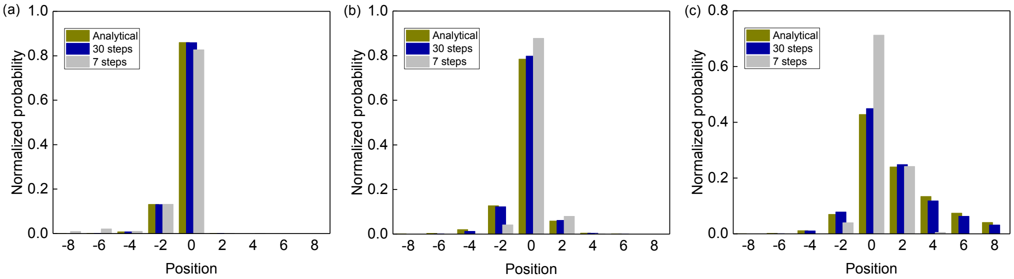

FIG. S3. **Confirming edge-state wave functions with numerical simulations.** We compare normalized probability distributions calculated from analytical edge-state wave functions (cyan) with those from numerical simulations after seven (grey) and thirty (blue) time steps. (a) Topological edge states between $\mathcal{PT}$-symmetry-unbroken bulks, with the same coin parameters and initial states as those of Fig. 2(b) in the main text. (b) Topological edge states between a $\mathcal{PT}$-symmetry-unbroken bulk and a broken one. Coin parameters and initial states as the same as those of Fig. 3(b) in the main text. (c) Topological edge states between $\mathcal{PT}$-symmetry-broken bulks. Coin parameters and initial states as the same as those of Fig. 3(c) in the main text.

the corresponding coin parameters $\theta^{\mathrm{L}}_{1,2}$ ($\theta^{\mathrm{R}}_{1,2}$), as well as the the quasienergy of the edge state $E^{(0,\pi)}$. The edge-state spatial wave function is therefore typically asymmetric with respect to the boundary.

On the other hand, $\bar{U}'$ has chiral symmetry with the symmetry operator $\Gamma=\sum_x |x\rangle\langle x|\otimes\sigma_x$ and $\Gamma\bar{U}'\Gamma=\bar{U}'^{-1}$. Edge states are therefore eigenstates of the chiral operator, such that $\frac{a_{-i\kappa_{\mathrm{L}}}}{b_{-i\kappa_{\mathrm{L}}}}=\frac{a_{i\kappa_{\mathrm{R}}}}{b_{i\kappa_{\mathrm{R}}}}=\pm1$. Equivalently, the edge states are either in $|+\rangle$ or $|-\rangle$. Combining the expressions of a_k and b_k, we derive conditions for the coin states. Specifically, edge states with quasienergy $E^{(0,\pi)}$ are in $|+\rangle$ when

$$\begin{cases}\sinh 2\kappa_{\mathrm{L}}=\frac{\cos E^{(0,\pi)}\sin\theta^{\mathrm{L}}_1+\sin\theta^{\mathrm{L}}_2}{\cos\theta^{\mathrm{L}}_1\cos\theta^{\mathrm{L}}_2},\\ \sinh 2\kappa_{\mathrm{R}}=-\frac{\cos E^{(0,\pi)}\sin\theta^{\mathrm{R}}_1+\sin\theta^{\mathrm{R}}_2}{\cos\theta^{\mathrm{R}}_1\cos\theta^{\mathrm{R}}_2}.\end{cases}\tag{S25}$$

And edge states are in $|-\rangle$ when

$$\begin{cases}\sinh 2\kappa_{\mathrm{L}}=-\frac{\cos E^{(0,\pi)}\sin\theta^{\mathrm{L}}_1+\sin\theta^{\mathrm{L}}_2}{\cos\theta^{\mathrm{L}}_1\cos\theta^{\mathrm{L}}_2},\\ \sinh 2\kappa_{\mathrm{R}}=\frac{\cos E^{(0,\pi)}\sin\theta^{\mathrm{R}}_1+\sin\theta^{\mathrm{R}}_2}{\cos\theta^{\mathrm{R}}_1\cos\theta^{\mathrm{R}}_2}.\end{cases}\tag{S26}$$

Finally, we show how to solve for the coefficients $r^{\mathrm{o(e)}}$ and $t^{\mathrm{o(e)}}$ by considering the Floquet operator $\bar{U}''=GF$. As discussed in Ref. [5], $\bar{U}''$ has chiral symmetry and support topological edge state at boundaries between regions with different topological numbers. Further, we notice that $\bar{U}'(\theta^{\xi}_1,\theta^{\xi}_2)=\bar{U}''(\theta^{\xi}_2,\theta^{\xi}_1)$, and that Eqs. (S25) and (S26) acquire different signs on exchanging θ^{ξ}_1 and θ^{ξ}_2. We then establish that edge states under $\bar{U}'$ and $\bar{U}''$ have the same coin states at $E^{(0)}$ ($\bar{\lambda}=1$), and they have opposite coin states at $E^{(\pi)}$ ($\bar{\lambda}=-1$).

On the other hand, for topological edge states $|\psi^{\mathrm{o(e)}}\rangle$ satisfying $\bar{U}'|\psi^{\mathrm{o(e)}}\rangle=\pm|\psi^{\mathrm{o(e)}}\rangle$, we have $\bar{U}''G|\psi^{\mathrm{o(e)}}\rangle=\pm G|\psi^{\mathrm{o(e)}}\rangle$. Therefore, $G|\psi^{\mathrm{o(e)}}\rangle$ is the edge state of $\bar{U}''$ with eigenvalues $\bar{\lambda}=\pm1$. By matching coin states of $|\psi^{\mathrm{o(e)}}\rangle$ and $G|\psi^{\mathrm{o(e)}}\rangle$ according to Eqs. (S21), (S25), and (S26), we derive the ratio $r^{\mathrm{o(e)}}/t^{\mathrm{o(e)}}$. Combining the normalization condition $\langle\psi^{\mathrm{o(e)}}|\psi^{\mathrm{o(e)}}\rangle=1$, we can solve for analytical expressions for the coefficients $r^{\mathrm{o(e)}}$, and $t^{\mathrm{o(e)}}$.

As a concrete example, we consider the case $(\theta^L_1,\theta^L_2)=(\pi/16,5\pi/16)$ and $(\theta^R_1,\theta^R_2)=(-9\pi/16,-5\pi/16)$, and derive the analytical wave function of the topological edge state with quasienergy $E^{(0)}$ on odd sites. According to Eqs. (S25) and (S26), coin states of $|\psi^o\rangle$ and $G\,|\psi^o\rangle$ are both $|+\rangle$. We then have

$$\begin{cases}\frac{\sqrt{B}t^o}{\sqrt{A}r^o}=\frac{(\cos\frac{\theta^L_1}{2}+\sin\frac{\theta^L_1}{2})(\cos\frac{\theta^R_2}{2}+\sin\frac{\theta^R_2}{2})}{(\cos\frac{\theta^R_1}{2}-\sin\frac{\theta^R_1}{2})(\cos\frac{\theta^R_2}{2}-\sin\frac{\theta^R_2}{2})}:=\tan\alpha,\\ \frac{(\sqrt{A}r^o)^2}{1-A^2}+\frac{(\sqrt{B}t^o)^2}{1-B^2}=\frac{1}{2},\end{cases}\Rightarrow\begin{cases}r^o=\sqrt{\frac{1-A^2}{2A}}\cos\alpha',\\ t^o=\sqrt{\frac{1-B^2}{2B}}\sin\alpha',\end{cases}\tag{S27}$$

where $A=e^{-2\kappa_L}$, $B=e^{-2\kappa_R}$, $\alpha'=\arctan(\sqrt{\frac{1-A^2}{1-B^2}}\tan\alpha)$, and $\alpha\in(-\pi/2,\pi/2)$. The analytical solutions of the edge-state wave function agree well with numerical results.

Edge-state wave function for non-unitary QWs

Consider topological edge states of $\bar{U}'$, with $\bar{U}'|\psi^{\mathrm{o(e)}}\rangle = \bar{\lambda}|\psi^{\mathrm{o(e)}}\rangle$ ($\bar{\lambda} = \pm 1$). Applying the non-unitary Floquet operator, we have

$$\tilde{U}'|\psi^{\mathrm{o(e)}}\rangle = F\gamma MG|\psi^{\mathrm{o(e)}}\rangle = \begin{cases} \gamma\,|\psi^{\mathrm{o(e)}}\rangle\,, \text{ if } \bar{\lambda}=1 \text{ and } |\psi^{\mathrm{o(e)}}\rangle \text{ in coin state } |+\rangle\,, \\ \frac{1}{\gamma}\,|\psi^{\mathrm{o(e)}}\rangle\,, \text{ if } \bar{\lambda}=1 \text{ and } |\psi^{\mathrm{o(e)}}\rangle \text{ in coin state } |-\rangle\,, \\ -\gamma\,|\psi^{\mathrm{o(e)}}\rangle\,, \text{ if } \bar{\lambda}=-1 \text{ and } |\psi^{\mathrm{o(e)}}\rangle \text{ in coin state } |-\rangle\,, \\ -\frac{1}{\gamma}\,|\psi^{\mathrm{o(e)}}\rangle\,, \text{ if } \bar{\lambda}=-1 \text{ and } |\psi^{\mathrm{o(e)}}\rangle \text{ in coin state } |+\rangle\,. \end{cases} \tag{S28}$$

Therefore, localized edge state of $\bar{U}'$ are also eigenstates of $\tilde{U}'$, with eigenvalues being $\pm\gamma$ or $\pm 1/\gamma$ in the non-unitary case. The corresponding quasiernergies satisfy $\mathrm{Re}(\epsilon) = 0, \pi$, which are exactly the conditions for topological edge states as required by pseudo-anti-unitarity of $\tilde{U}'$ [6]. We therefore conclude that topological edge states under $\tilde{U}'$ have the same spatial and coin-state wave functions as those in unitary case. The difference lies in the quasienergies and hence the time evolution.

For edge states with eigenvalues $\pm\gamma$, the corresponding quasienergies are $i\ln\gamma$ and $\pi + i\ln\gamma$, respectively. Their probability distributions increase during the time evolution as γ^{2t}, and we identify them as the bright edge states. In contrast, for edge states with eigenvalues $\pm 1/\gamma$, the corresponding quasienergies are $-i\ln\gamma$ and $\pi - i\ln\gamma$, respectively. Their probability distributions decrease during the time evolution as γ^{-2t}, and we identify them as the dark edge states.

Due to $\mathcal{PT}$ symmetry of $\tilde{U}'$, eigenstates with the eigenvalues λ and λ^{-1} must appear in pairs. This implies that bright and dark edge states must also appear in pairs. From Eqs. (S25) and (S26), we see that at a given boundary, edges states associated with the same topological number (ν_0 or ν_π) have the same coin states and are of the same type (bright or dark). In fact, they only differ by the occupation of odd or even sites. Thus, edge states associated with the same topological number are two-fold degenerate. Further, bright edge states at a given boundary should change into dark ones, and vice versa, when we exchange the coin parameters on the two sides of the boundary. All these conclusions are consistent with numerical calculations, where $\tilde{U}'$ is directly diagonalized.

Finally, we confirm the analytical edge-state wave functions derived above by comparing the normalized probability distributions from the analytical solution and from numerical simulations of QW dynamics governed by $\tilde{U}'$. In Fig. S3, we see that as the time steps of the numerical simulation increase, the resulting normalized probability approaches that of the analytical solution. Apparently, it takes more time steps for the QW dynamics to converge to the edge-state distribution when at least one of the bulks is $\mathcal{PT}$-symmetry broken.

[1] Xiao L, Zhan X and Bian ZH *et al.* Observation of topological edge states in parity–time-symmetric quantum walks. *Nat. Phys.* 2017; **13**: 1117-1123.

[2] Mochizuki K, Kim D. and Kawakami N *et al.* Bulk-edge correspondence in nonunitary Floquet systems with chiral symmetry. *Phys. Rev. A* 2020; **102**: 062202 (2020).

[3] Zeuner JM, Rechtsman MC and Plotnik Y *et al.* Observation of a topological transition in the bulk of a non-Hermitian system. *Phys. Rev. Lett.* 2015; **115**: 040402.

[4] Zhan X, Xiao L and Bian Z *et al.* Detecting topological invariants in nonunitary discrete-time quantum walks. *Phys. Rev. Lett.* 2017; **119**: 130501.

[5] Asbóth JK and Obuse H. Bulk-boundary correspondence for chiral symmetric quantum walks. *Phys. Rev. B* 2013; **88**: 121406(R).

[6] Esaki K, Sato M and Hasebe K *et al.* Edge states and topological phases in non-Hermitian systems. *Phys. Rev. B* 2011; **84**: 205128.
